# Investigating the role of obesity, circadian disturbances and lifestyle factors in people with schizophrenia and bipolar disorder: Study protocol for the SOMBER trial

**DOI:** 10.1371/journal.pone.0306408

**Published:** 2024-07-08

**Authors:** Mikkel EI Kolind, Rikke Kruse, Anni S. Petersen, Charlotte S. Larsen, Lasse K. Bak, Kurt Højlund, Christoph P. Beier, Elsebeth Stenager, Claus B. Juhl

**Affiliations:** 1 Steno Diabetes Center Odense, Odense University Hospital, Odense, Denmark; 2 Department of Regional Health Research, University of Southern Denmark, Odense, Denmark; 3 Open Patient data Explorative Network—OPEN, University of Southern Denmark, Odense, Denmark; 4 Department of Endocrinology, Hospital of South West Jutland, Esbjerg, Denmark; 5 Department of Clinical Biochemistry, Copenhagen University Hospital, Rigshospitalet, Glostrup, Denmark; 6 Department of Clinical Research, University of Southern Denmark, Odense, Denmark; 7 Department of Neurology, Odense University Hospital, Odense, Denmark; Arba Minch University, ETHIOPIA

## Abstract

The aim of this study is to investigate circadian rhythms in independently living adults with obesity and mental disease, exploring the interplay between biological markers and lifestyle factors. Eighty participants divided equally into four groups; (i) people with obesity and schizophrenia; (ii) people with obesity and bipolar disorder; (iii) people with obesity without mental disease or sleep disorders, and (iv) people without obesity, mental disease or sleep disorders. Over two consecutive days, participants engage in repeated self-sampling of hair follicle and saliva; concurrently, data is collected on diet, body temperature, light exposure, sleep parameters, and physical activity by accelerometry. Hair follicles are analyzed for circadian gene expression, saliva samples for cortisol and melatonin concentrations. Circadian rhythms are investigated by cosinor analysis. The study employs a participant-tailored sampling schedule to minimize disruptions to daily routine and enhance ecological validity. The methodology aims to provide a comprehensive insight into the factors contributing to circadian disruptions in people with obesity, bipolar disorder and schizophrenia, potentially informing strategies for future management and mitigation.

**Trial registration:** (ClinicalTrials.gov Identifier: NCT05413486).

## Introduction

**Fig 1:**
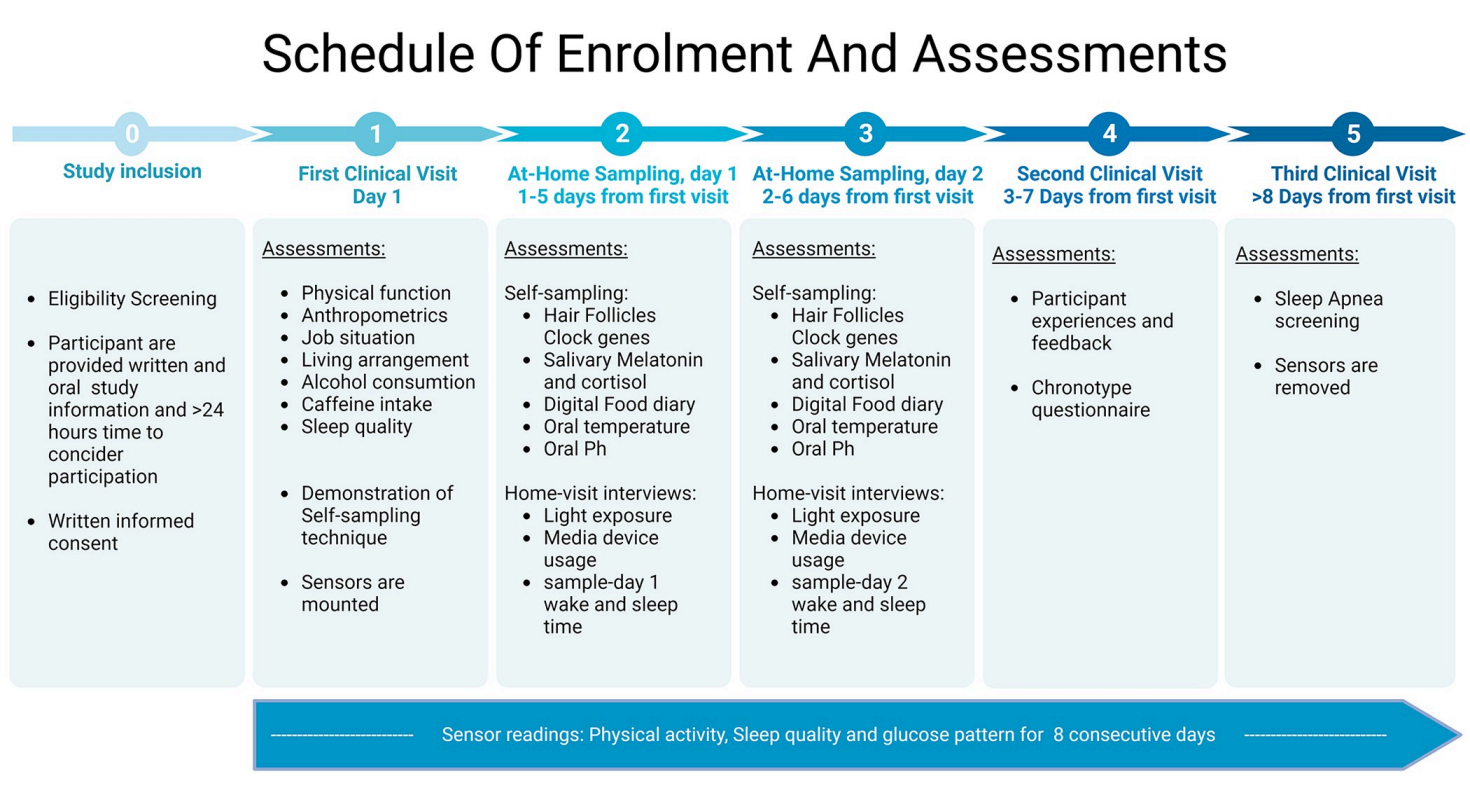
Schedule of enrolment and assessments at all time points in the SOMBER Trial.

This study protocol provides a comprehensive overview of the study procedures and methods in the Sleep, Obesity and Mental Disease—Biological Markers for the Evaluation of Circadian Rhythmicity (SOMBER) trial (ClinicalTrials.gov Identifier: NCT05413486). The study procedure for the SOMBER trial is outlined in Fig 1.

### Background

Circadian disturbances have emerged as a significant health concern with profound implications for human well-being. From endocrine [1], cardiovascular [2] and immune function [3] to energy homeostasis, digestion, metabolism [4] and cell signalling [5], practically all aspects of human physiology are affected by the relative time-of-day in relation to the natural human sleep-wake cycle. Modern-day societal norms and advancements in technology have led to widespread disruptions of the circadian rhythm: Night time light pollution [6], shift work, irregular sleep schedules and jet lag all contribute to these disruptions [7]. Circadian misalignments are known to contribute to a variety of psychiatric disorders, creating a bidirectional relationship, where mental health issues can predispose individuals to poor sleep quality, which in turn may exacerbate symptoms of the underlying disorder, such as heightened anxiety and increased rumination. This cycle can further complicate sleep patterns, making it more challenging to achieve restful sleep [7,8]. Disruptions to the circadian cycle not only aggravate the symptoms of mental diseases but likely also play a significant role in triggering first symptoms at onset [9].

The suprachiasmatic nuclei (SCN) are the brain’s central circadian pacemakers primarily entrained through non-visual photosensitive retinal ganglion cells when sun light hits the retina. Human circadian rhythms are thus aligned with the light-dark cycle. Even in the absence of light stimulus, this rhythm is maintained in part by so-called clock genes, which generate self-sustaining circadian oscillation through positive and negative transcriptional/translational feedback loops. Clock genes work both in concert with and independently of the SCN [10] and multiple diseases are linked to abnormal clock gene function.

In schizophrenia (SCH), clock gene disruption has been demonstrated for *CRY1* and *PER2* in skin fibroblasts, *PER1/2/3* and *NPAS2* in white blood cells, and in post-mortem analyses for P*ER1/2*, *CRY2*, and *NR1D1* in the prefrontal cortex [11–13]. Similarly, in bipolar disorder (BD), whole blood analysis have revealed reduced expression of *CRY 1/2*, *PER 1/2/3* and *ARNTL* during depressive episodes [14]. Similar results have been reported for clock genes in skin fibroblast [15].

**Fig 2:**
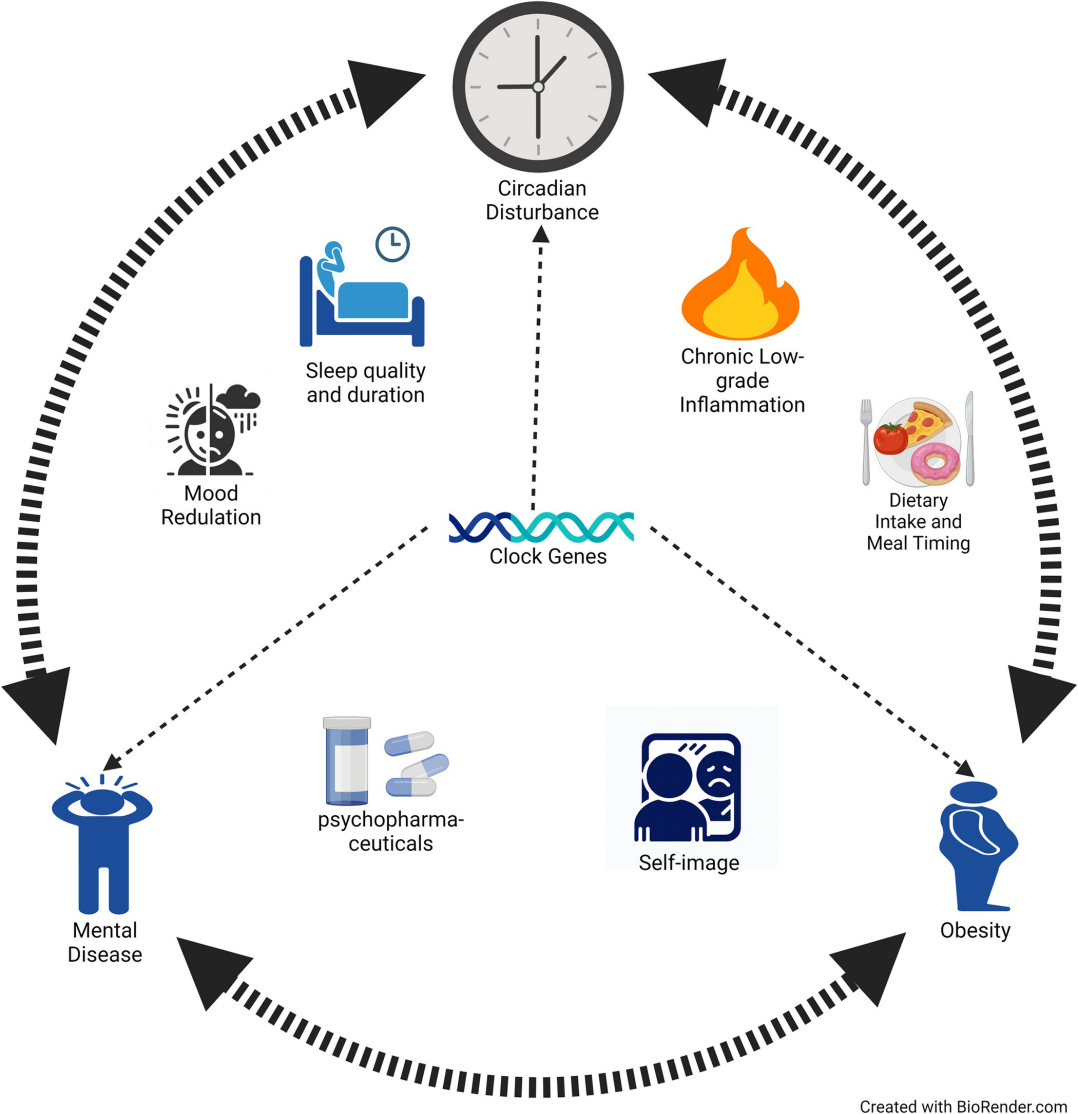
The tridirectional relationship between circadian disturbances, mental disease and obesity. Circadian disturbances, mental disease and obesity are associated through a complex network of intermediating factors, each potentially influencing or being influenced by other factors in the network.

A common denominator of both mental disease and circadian disturbances is the link with obesity [16,17]. Circadian disturbances, mental disease and obesity seems to form a tridirectional relationship where symptoms or condition-related disease severity may alter the progression of either one or both of the other two conditions. For example, in schizophrenia, anti-psychotic medicine often lead to weight gain and increased obesity risk [18]. Obesity in turn increases the risk of sleep disturbances (e.g., poor sleep quality and/or obstructive sleep apnoea (OSA)) [19]. Sleep disturbances may then both make the patient more susceptible to emotional instability [20] as well as obesity e.g., by triggering emotional eating or leading to increased night-time snacking [16]. Meal timing, diet, social cues, media device usage, and light exposure are just some of the factors influencing, and influenced by, circadian disruptions, which may in turn lead to obesity [21]. Both obesity and schizophrenia are associated with increased levels of tumor necrosis factor-α and other pro-inflammatory cytokines with established impact on the physiological regulation of sleep [19,22]. These cytokines ordinarily follow a circadian rhythm, which may be disturbed in people with obesity and sleep disorders [19]. In addition, several clock genes commonly disrupted in schizophrenia have been shown to play a key role in energy and lipid metabolism [23–25]. Altogether, the tridirectional relationship between circadian disturbances, mental disease and obesity is complex and consist of several interloping vicious cycles. Fig 2 presents some examples of intermediate pathways between circadian disturbances, mental disease and obesity.

Circadian rhythms in humans can be evaluated through various biomarkers, including hormonal peak values and rhythms, daily body temperature changes, and rhythmic gene expression [26]. Since circadian rhythms are closely intertwined with daily routines, measurements taken in laboratories or in-hospital settings may have low ecological validity due to the impact of these artificial environments. Therefore, there is a need for minimally invasive sampling techniques, compatible with daily routines, which can be applied in a real-life setting.

The primary aims of this study are to: (i) To investigate biological markers of disturbed circadian rhythm in free-living adults with obesity and schizophrenia or BD using non-invasive methods and; (ii) to compare biological markers of disturbed circadian rhythm with subjective and objective measures of sleep quality.

## Methods

This is a case-control study with repeated measures. We will enrol a total of 80 participants, across four different study groups: (i) People with obesity (BMI ≥ 30 kg/m^2^) and schizophrenia spectrum disorder (F20-29) (OB+SCH, n = 20); (ii) people with obesity and bipolar spectrum disorder (F30-31) (OB+BD, n = 20); (iii) people with obesity without mental disease or (non-OSA) sleep disorders (OB, n = 20) and (iv) people with normal BMI (18–25 kg/m^2^) and no mental disease or sleep disorders as control group (CON, n = 20). OB+SCH are matched with OB and CON on gender, age, length of day, BMI, and sleep apnoea. OB+SCH is additionally matched with OB on BMI and presence or absence of OSA. Due to a limited recruitment pool of participants with BD, these participants are not matched (Table 1).

**Table 1:** Group matching and exclusion criteria.

	Obesity and schizophrenia (n = 20)	Obesity and bipolar disorder (n = 20)	Obesity and no mental disease (n = 20)	Non-obese control group (n = 20)
*Excluded if*	• Use of oral melatonin	• Use of oral melatonin	• Any sleep medication	• Any sleep medication
			• Any sleep disorder, except sleep apnoea	• Any sleep disorder
			• Any psychiatric disorder	Any psychiatric disorder
*Matched on*	✓ Gender (male/female)	unmatched	✓ Gender (male/female)	✓ Gender (male/female)
	✓ age (± 5 years)		✓ age (± 5 years)	✓ age (± 5 years)
	✓ time of year (± 21 day)		✓ time of year (± 21 day)	✓ time of year (± 21 day)
	✓ sleep apnoea (yes/no)		✓ sleep apnoea (yes/no)	Not applicable
	✓ BMI (± 3 kg/m^2^)		✓ BMI (± 3 kg/m^2^)	Not applicable

### Recruitment

Participants are primarily recruited at the obesity clinic at the University Hospital, Southern Denmark, Esbjerg and through local out-patient psychiatric treatment units and patient networks. Psychiatric out-patients are referred to the study by health professionals aware of the trial design. Additional non-obese controls are recruited through online postings on social media. Recruitment started April 2022 and is scheduled to complete by August 2024. All study procedures are in adherence with the principles of the Declaration of Helsinki and approved by the Regional Health Ethics Committee (S-20210181 with amendments). Written Informed consent was obtained from all participants. The investigator is responsible for obtaining informed consent and enrolling patients in the study.

### Study procedure

Participants will visit the clinic three times. Between clinical visit 1 and 2, participants will collect saliva and hair follicles over a 2-day period. In addition, participants will wear accelerometers and continuous glucose monitors (CGMs) for a total of 8 days. The study procedure is outlined in Fig 1 and detailed chronologically below.

#### Clinical visit 1

During the first clinical visit, data will be gathered on anthropometry (height, weight, body composition (by bioimpedance (InBody770®, InBody, Seoul, Korea)), hip and waist circumference), physical function (grip strength (by hand dynamometer (Kern & Sohn, Balingen, Germany)), 10 m usual and maximal gait speed, 1-leg standing balance) and patient-reported outcomes (intake of caffeinated drinks, alcohol consumption, habitual use of sleep medications, socioeconomic status, living arrangements, education, ethnicity, hair-colour, co-morbidities and sleep quality (PSQI [27]).

**Fig 3:**
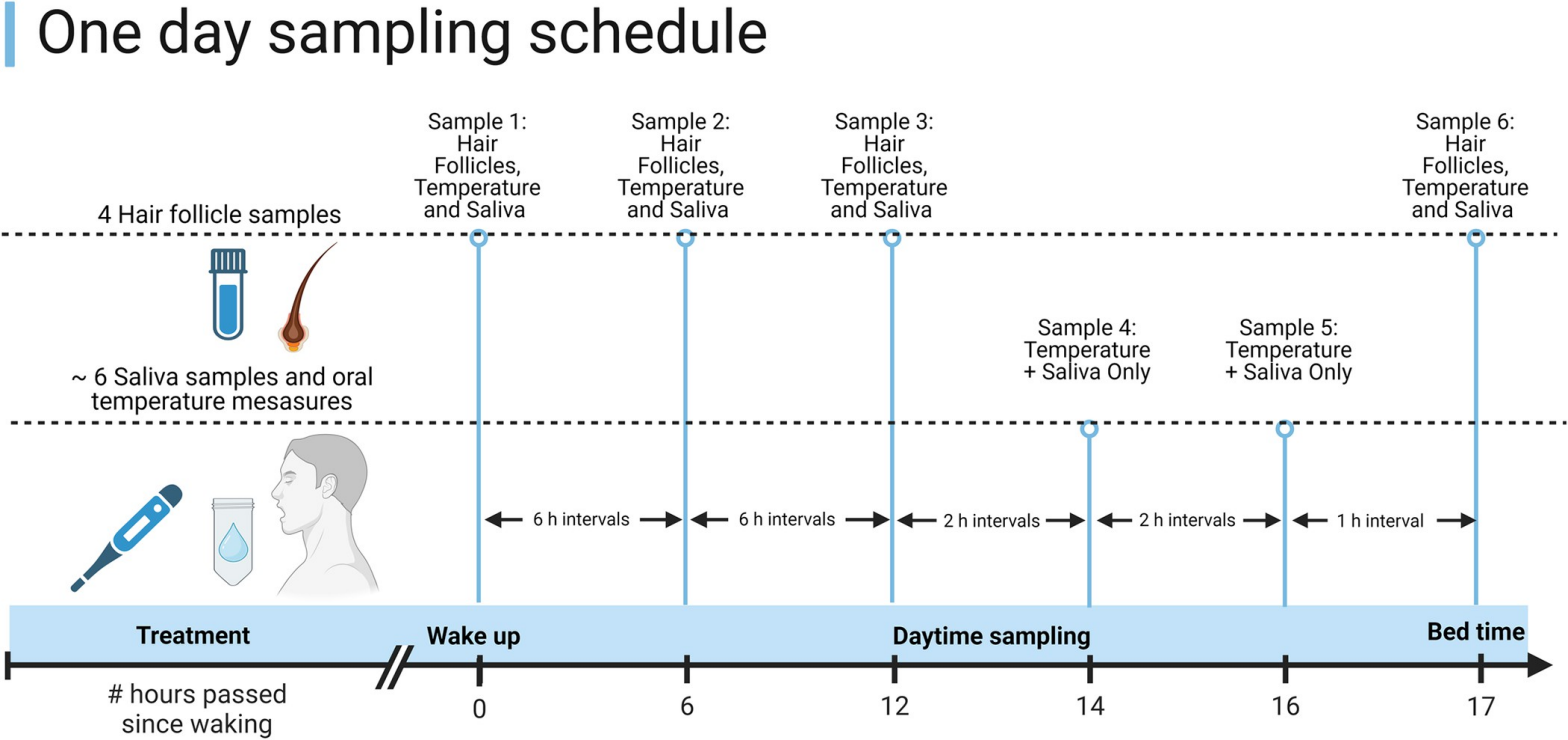
Example of sampling schedule. Above schedule assumes a wakefulness period of seventeen hours. Schedules are adjusted relative to individual rhythms. Participants are instructed to start sampling of hair follicles and saliva at awakening and every six hours hereafter, as well as one sample at bed time. Additional saliva samples are collected every two hours after twelve hours wakefulness and approximately one hour before bedtime. Oral temperature is measured concurrently with saliva sampling.

After initial testing, accelerometers (Axivity AX3, Axivity Ltd., Newcastle upon Tyne, United Kingdom) and CGMs (Freestyle libre-2, Abbott Laboratories, Lake Bluff, Illinois, USA) are mounted and the participant is given thorough instruction on how to record dietary intake and self-sample (See “data collection” below). Self-sampling will be performed at the time points outlined in Fig 3. Participants are provided with all necessary equipment (sampling tubes, manuals, camera, tweezers and scissors). The two sampling days will be placed within 1–5 days of the first clinical visit. Importantly, participants are asked to choose two days with an ordinary daily rhythm, especially in regards to sleep and wake time and to avoid irregular days (e.g., celebratory events or holidays). Manuals, protocols and trial-specific questionnaires utilized during clinical visit 1 are available as (S1–S3 Files).

#### At-home testing

For two consecutive days participants will sample saliva approximately six times (depended on wakefulness period) per day, hair samples four times per day and record body temperature and dietary food intake (S1 File). Participants are provided with an individualized schedule indicating all sampling times. Participants will collect saliva by a chewable saliva collector (Salivette Cortisol saliva collector, Sarstedt, Nümbrect, Germany). Saliva sampling entails placing one (or two in case of xerostomia, common with some psychotropic drug [28]) small moisture absorbing cottonous tube in the mouth which is then spat into a test tube and refrigerated. Participants are instructed to avoid food or drinks 30 minutes before saliva sampling, and to follow their normal meal times. Oral temperature are collected simultaneously with saliva sampling using an oral thermometer according to manufacturers’ instructions (Apotekets, Skovlunde, Denmark). Hair follicles are self-sampled with tweezers. Participants will pluck 20 hairs from the scalp or chin and place these in a date- and time marked tube containing dissolution buffer (RNeasy Micro Kit; QIAGEN, Hilden, Germany). Immediately after sampling, follicle- and saliva samples are placed in the refrigerator. If participants need to collect samples away from their home they are provided with cooling bags and icepacks.

During each of the two home-sampling days, participants are offered home visits from a study representative, a health professional trained in collecting hair follicles. If the participant declines this offer, telephone meetings are scheduled instead. If possible, home visits will be scheduled around a sampling time, during these visits the sample is collected by the study representative. This is to allow for comparison between self- and professional- sampling. During visits, additional data is gathered on light exposure and media device screen time usage (S4 File), and any potential safety concerns are noted and dealt with as applicable under the regulations for out-patient treatment.

#### Clinical visit 2

Following the two sampling days, participants will again meet at the clinic. Participants are asked to fill in the Morningness-Eveningness Questionnaire (MEQ) and a brief questionnaire on their experience on how easy/troublesome they found different aspects of sampling.

#### Clinical visit 3

At the final visit, a time for at-home sleep apnoea screening is scheduled. Sleep apnoea is tested for using a Nox-T3 sleep apnoea screening kit (Nox Medical, Reykjavik, Iceland). As this last visit only requires the participant to receive a 5-minute introduction in using the NOX-T3 equipment and dropping off accelerometers, this visit may instead be handled as a home visit. To avoid unnecessary tests, participants with recent (<6 months) apnoea screening available from registries are not re-assessed, unless there is reason to suspect a change in apnoea status, e.g., excessive weight loss/gain or change in medication.

#### Information from patient registries

Supplementary information is collected from patient registries. This includes use of prescription medicine, active diagnoses, health and disease screenings, blood samples and questionnaire responses, of note the Epworth Sleepiness Scale [29] and the Berlin Questionnaire, both sleep related questionnaires used to screen for sleep apnoea [30].

A summary schedule of study outcomes is presented in Table 2.

**Table 2:** Summary of all data gathered at all time points and from registries.

	Clinical visit 1	Home visit 1	Home visit 2	Clinical visit 2+3	From patient registries
*Data gathered*	• Anthropometrics • Physical function	• Clock gene expression• Cortisol rhythm• Melatonin rhythm• Food Diary• Oral temperature• Oral Ph	• Clock gene expression• Cortisol rhythm• Melatonin rhythm• Food Diary• Oral temperature• Oral Ph	• Sleep apnoea screening	• use of prescription medicine • Lipid and hormonal blood profiles • body composition
	Questionnaire data: • Sleep Quality • Oral sleep aids • Daily coffee and alcohol intake • Job situation • Living arrangement	Questionnaire data: • light exposure • screen time • sample-day 1 wake and sleep time	Questionnaire data: • light exposure • screen time • sample-day 2 wake and sleep time	Questionnaire data: • Participant feedback • Chronotype	Questionnaire data: • Tiredness and sleep • Lifestyle • Habits • Pain
	Continuous objective measures (8 days, starting clinical visit 1): • Sleep amount and quality by accelerometer • Skin surface temperature changes by accelerometer • Daily glucose patterns (by continuous glucose monitor)				

#### Patient monitoring and safeguards

Throughout the sampling period, we maintain regular contact with participants to promptly address any signs of undue stress, discomfort, or negative health findings. Our protocol includes daily follow-up communications on sampling days. Should any adverse events or critical health findings arise, early termination may be considered, and participants may be directed to appropriate health treatment facilities for further evaluation or treatment.

### Laboratory analyses

All laboratory tests are performed by researchers blinded to all other outcomes and group allocations.

#### Saliva hormonal analyses

Within 24–72 hours after sampling, samples are centrifuged at 2000g for 5 minutes. Immediately afterward, they are stored at -80°C until analyzed for cortisol, melatonin, and pH at the Department of Clinical Biochemistry, Copenhagen University Hospital, Rigshospitalet, Glostrup, Denmark. For cortisol analysis, 200 μl of saliva is analyzed using an IDS-iSYS Multi-Discipline Automated System (Immunodiagnostic Systems, Tyne & Wear, United Kingdom) with the CE-labeled Salivary Cortisol kit (IS-4900). Where possible, the pH of the saliva samples analyzed for cortisol is measured using a standard pH electrode (Mettler-Toledo FiveEasy, Leicester, United Kingdom); samples for pH measurement undergo one additional freeze-thaw cycle before analysis. Melatonin is quantified using 2x50 μl of saliva with the CE-labeled Saliva ELISA kit (IBL International, Hamburg, Germany), in combination with a standard ELISA reader (Perkin Elmer 1420 Victor 3 Multi Label Counter; Shelton, Connecticut, USA). If the saliva volume is insufficient for both cortisol and melatonin analyses, cortisol is prioritized.

#### Hair follicle clock gene expression

All hair follicles are analyzed at Steno Diabetes Center Odense, Odense, Denmark. After collection during home visits the samples are stored at -80° C until further analysis. RNA from the hair follicles is isolated using an RNeasy Micro kit (QIAGEN, Hilden, Germany) according to the manufacturer’s instructions. Briefly, the samples are thawed on ice after which they are centrifuged to precipitate the hair follicle cells. After removing the supernatant, the cells are lysed using a RLT buffer supplemented with DTT. The lysates are then processed through spin-columns, treated with DNase (Qiagen, Denmark) to remove genomic DNA, washed with RW1 and RPE buffers, and subjected to ethanol precipitation. The final RNA is then eluted in a minimal volume of RNase-free water and quantified by NanoDrop spectrophotometer (Thermo Fisher Scientific, Waltham, MA). RNA is reverse transcribed to cDNA using a high-capacity cDNA reverse transcription kit (Applied Biosystems, Foster City, CA) according to manufacturer’s protocol. The qPCR is performed on a QuantStudio 5 real-time PCR system (Applied Biosystems, Foster City, CA) using the pre-designed commercially available TaqMan Custom Arrays (Applied Biosystems, Foster City, CA). All samples are run in triplicates. We will test the gene expression of a panel of circadian rhytm-related genes (e.g. *PER3*, *NR1D1/2*). The expression of any genes of interest will be normalized to appropriate reference genes. We will test a panel of reference genes to identify the most appropriate and stable candidates.

### Data handling and analyses

Data will be hosted and handled in accordance with Danish law and regulation. The trial dataset is accessed by the investigators (MEIK and CBJ). MEIK will perform the data analyses and takes responsibility for the data integrity. All data files are retained for 5 years after the study’s conclusion, after which they will be either anonymized or deleted.

#### Main outcomes

The primary outcome is circadian expression of clock-genes, the secondary outcomes are circadian salivary cortisol and melatonin rhythms.

#### Power calculation

Based on Sun et al. (2016) [12], and targeting a statistical power of 0.8 and an alpha error of 0.05, our study requires groups of 17 participants for the mesor and 16 participants for the amplitude to show statistically significant group differences in clock gene expression, as measured by unpaired t-tests.

#### Statistical analyses

The four observational groups will be compared on group characteristics of e.g., BMI, body composition, physical function and SES using One-Way Analysis of Variance (ANOVA) or Kruskal-Wallis tests for continuous or ordinal outcomes, and Chi-squared statistics for categorical outcomes as dictated by model assumptions and applying suitable post-hoc analyses as appropriate.

The primary outcome will be analyzed for each study group. Rhythmicity will be assessed by approximating each individual’s 48-hour profiles to a 24 hour cosine wave function in non-linear mixed effects models thereby allowing for the estimation of amplitude, acrophase, and mesor for each group. Derived variables will be compared between groups using the non-obese control group as the reference. Inter-day variability of circadian parameters will be analyzed for each group. Cosine analyses will be individualized relative to participants objectively measured mean sleep offset time.

The same procedure will be used for the secondary measures of rhythmicity i.e. salivary cortisol and melatonin values. In addition, peak values and peak to trough values will be calculated and compared between groups. Wake-to-bed slopes of cortisol will be calculated by subtracting the bedtime sample value from the sample collected immediately upon awakening and divided by the number of hours separating these two samples [31]. Dim light melatonin onset (DLMO) is estimated by comparing evening melatonin concentrations to the mean levels obtained from samples collected during daylight hours. Other potentially rhythmic variables (blood glucose, oral pH and oral temperature) will be visually reviewed for rhythmicity and if applicable analyzed by similar methods.

The correlation between clock gene expression and sleep parameters (e.g., sleep quality, MEQ-category, AHI, and other sleep parameters (S5 File)) will be investigated through Spearman’s rho in correlation matrices. Additional potentially confounding variables will be added to these matrices. This includes variables related to diet, body composition, medication, physical activity and media device usage.

Significant findings from the correlational matrices will be further evaluated by longitudinal analyses (e.g. linear regression). In these analyses, measures of circadian rhythmicity e.g., acrophase of clock gene expression, will be used as dependent variables. Independent variables will be ‘group allocation’, BMI, gender and age. Variables with an insignificant effect will be skipped from the model. Subsequently, other variables with a potentially confounding influence as identified in the correlational matrix will be added to the model. The most appropriate model will be selected based on the Akaike Information Criterion (AIC), with preference given to the model having the lowest AIC score [32].

In all tests, a p-value below 0.05 is considered significant.

## Discussion

This study has the potential to significantly advance circadian rhythm assessment by implementing multiple self-sampling techniques in Scandinavian cohorts of people with diverse mental health statuses and varying degrees of obesity. Hair follicle sampling, as highlighted in a recent literature review by Crnko et al. (2021), offers high-quality RNA extraction with minimal invasiveness, presenting a promising technique for clock gene expression analysis [26]. The application of this method in a Scandinavian demographic is particularly notable, as previous research has primarily concentrated on Asian populations, which exhibit hair characteristics distinct from Caucasian populations [33].

Furthermore, the comparative analysis of self- versus professionally-assisted sampling and collection of participant experiences within our study will yield comprehensive insights into the practicality of this method. Such information is vital to assess the wider applicability and feasibility of hair follicle sampling across various demographic groups.

Previous research has identified circadian disturbances, marked by altered clock gene expression, as factors in various somatic and mental diseases. These range from obesity [23–25], OSA [34], type-2 diabetes [35], leukemia [36], mood disorders [37], major depressive disorder (MDD), schizophrenia and BD [38–40]. Interestingly, in a recent study designed to evaluate cross-disorder polygenic risk scores for chronotype, sleep duration and insomnia across BD, MDD, and schizophrenia, there was a genetic overlap in all three disorders with each trait [41]. Despite this, no study to date have allowed for a direct group comparison of rhythmic clock gene expression between these diagnoses. Furthermore, no study has assessed the comorbid effect of obesity and mental disease on clock gene expression.

### Strength and limitations

The primary strength of our study lies in its minimally invasive, at-home sampling approach. This method allows participants to collect data on their own, with minimal interference to daily routines.

A notable limitation of our study is the relatively broad inclusion criteria for participants with mental health conditions. This inclusivity, while enriching the diversity of our sample, introduces variability in comorbidity, medication use and symptomatological characteristics among participants. Consequently, this heterogeneity may decrease our ability to detect significant effects and dilute the statistical power needed for potential subgroup analyses. All participants are outpatients primarily referred through psychiatric treatment units and user organizations by certified health professionals. Health professionals are aware of the study design and scope, and therefore, participants are expected to have stable symptoms and be deemed adequately cognitively fit to complete the entire study trial. While explicitly stating symptom stability and neurocognitive health as inclusion criteria would have added transparency to our trial, the recruitment process likely ensures that our study population resembles other studies with explicitly stated criteria. For example, Adan et al. (2024) excluded patients with induced disorders, those with unstable symptoms or not in remission, and those with conditions affecting the assessment process (i.e., neurological and neurocognitive disorders) [42]. Moreover, since symptom characteristics are not specifically measured, we cannot compare participants with predominant negative or positive symptomatology. Sampling schedules are tailored to each participant’s sleep-wake cycle, differing from the fixed schedules commonly used in hair follicle sampling studies [37,43,44]. This customization better aligns with participants’ usual rhythms, allowing those with varying sleep patterns to adhere to the schedule without altering their wake or bedtimes. This is especially important since participants in the OB+SCH and OB+BD groups are expected to have significantly later sleep onset and longer sleep periods [45,46]. Additionally, our schedule enables most employed participants (mainly in the CON and OB groups) to conveniently collect samples at wake-up, during lunch breaks, and after work hours.

Similarly, salivary sampling times in our study are chosen for participant compliance. Typically, cortisol samples are collected several times within the first hour after awakening to estimate the cortisol awakening response [31,47]. However, this would result in the participant having to postpone breakfast and would not align with a natural work rhythm. Similarly, for melatonin, we modified the standard Danish method of sampling every 30 minutes from a fixed evening time (e.g., 5 pm) until bedtime [48] to two-hour intervals to accommodate regular eating patterns, with an additional sample one hour before bedtime to enhance the accuracy of DLMO prediction.

Overall, the chosen sampling schedule for hair follicles and saliva are designed to capture major shifts in clock gene and hormonal rhythms due to circadian disruption or mental disease while disrupting daily habits as little as possible. This approach, while enhancing ecological validity of the study, introduces a significant degree of variability compared to more controlled clinical studies. To account for this variability, we are implementing measures to track and adjust for several confounding variables.

### Supplementary analyses

In addition to assessing clock gene expression and hormonal rhythms, we incorporated secondary indicators of circadian rhythms. Specifically, oral temperature and pH measures hold promise as potential cost-effective proxies for circadian rhythms [49]. Oral temperature readings are augmented by skin surface temperature data from the AX3 accelerometer’s built-in thermometer. Despite limited sensitivity, the AX3 has been shown to improve the precision of sleep registration [50], providing a valuable continuous record of skin surface body temperature changes. Finally, glucose levels as determined by CGM show distinct circadian rhythms [51]. The inclusion of this measure could facilitate new understanding of the relationship between glucose patterns and clock gene expression in people with mental disease.

### Confounding variables

Circadian rhythms are potentially affected by many factors such as light exposure, meal timing, media device usage and physical activity [21], the most powerful Zeitgeber is the absence or presence of sunlight. New recommendations suggest that light intensities exceeding even 1 lux (with respect to its melanopic equivalent daylight illuminance(MEDI) in the sleeping environment may negatively impact sleep quality and that MEDI should be kept >10 lux in the hours before sleep [52]. Use of LED-screened media devices presents a high proportion of short-wavelength light and have been shown to delay self-selected bedtime [53] and suppress melatonin [54]. Present protocol includes self-reported measures of light exposure and media device usage, thus allowing us to control for the exposures.

Dietary intake and timing are documented using a digital camera, while physical activity is monitored by accelerometer (thigh and wrist placement). Both diet and physical activity are known to influence sleep quality and the circadian rhythm [55,56]. Additionally, research has consistently shown variations in diet and physical activity patterns in relation to mental health conditions [57,58] and obesity [59]. By tracking these measures, we can refine our analyses and identify potential dietary outliers, enhancing the robustness of our study.

This comprehensive approach enables us to explore the intricate interplay between various factors and their impact on circadian rhythms. By integrating non-invasive methods to investigate biological markers of disturbed circadian rhythms in independently living adults with obesity and schizophrenia or BD, we aim to gain a holistic understanding of the factors that contribute to circadian disruptions for people in these groups. This, in turn, will provide valuable insights into the mechanisms underlying these disturbances and inform strategies for their management and mitigation.

## Dissemination policy

The findings of this research will contribute to a doctoral thesis. Additionally, we will share the outcomes at scientific conferences and seek publication in academic journals. Patient organizations will also be informed of our study results. We commit to transparently sharing all data, whether favorable or unfavorable.

## Supporting information

S1 File Handouts: Sampling guides and sampling schedules with translations. (PDF)

S2 File Clinical visit 1—Supplementary questionnaires with translations. (PDF)

S3 File Procedure for initializing and mounting AX3 accelerometers in the SOMBER trial. (PDF)

S4 File Home visit protocol and questionnaire. (PDF)

S5 File List of supplementary variables. (PDF)

S6 File SPIRIT-Outcomes 2022 checklist. (PDF)

S7 File Protocol revision history. (PDF)
