# Supplementary material for: Investigating the role of obesity, circadian disturbances and lifestyle factors in people with schizophrenia and bipolar disorder: Study protocol for the SOMBER trial
Source: PLoS One. 2024 Jul 8;19(7):e0306408. doi: 10.1371/journal.pone.0306408 (PMC11230533; doi:10.1371/journal.pone.0306408)
Supplement: S1 File — (PDF) [file pone.0306408.s001.pdf]

# Miniguide til prøvetagning

Døgnrytme-forstyrrelser hos personer med psykiske lidelser

<u>Bevægelsessensorer (accelerometrer)</u>

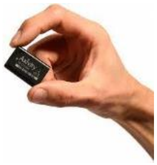

Bevægelsessensorerne måler dit søvnmønster og generelle fysiske aktivitetsniveau over syv døgn. De måler selv alt og kræver ikke nogen handling fra din side af. Det er vigtigt du ikke fjerner accelerometeret før de syv dage er gået.

Begge accelerometerere starter: d._____ / _____ kl:____ : _____ (starter automatisk)
Begge accelerometerere er færdige: d._____ / _____ kl:____ : _____ (og stopper automatisk)

<u>Glukosemåler</u>

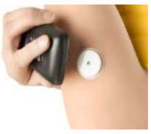

Glukosemåleren måler ændringer i dit blodsukker. **Den bør scannes ca. hver 6.time.** Vigtigst lige når du står op.

Glukosemåleren er sat i gang d._____ / _____ kl:____ : _____
Glukosemåleren er færdig d._____ / _____ kl:____ : _____ (skal måle samtidig med accelerometere)

<u>Hårprøver:</u>

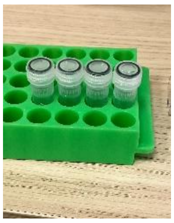

Når du tager hårprøver er det kun folliklen nær hårroden som indeholder mRNA. Det er derfor vigtigt at håret fjernes med så meget rod som muligt. Hvis du ikke kan se noget af den fedtede hinde omkring roden er håret sandsynligvis knækket af for højt og kan ikke bruges. Du skal plukke ca. 20 hår per prøve.
Når du har plukket et hår klippes hårroden fra ned i en af mRNA-rørene. Sørg for at den afklippede hårrod dækkes af væske. Det er kun hårroden som kan anvendes til analyse. Hårstammen skal ikke ned i væsken.

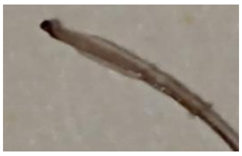

<u>Her kan du se hele hårroden, hvis intet af den fedtede hinde er synligt, er håret knækket for højt og du er nød til at tage en ny prøve</u>

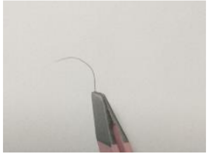

<u>Håret her ser ud til at være knækket for højt (ingen synlig hårrod) og kan desværre ikke bruges.</u>

<u>Spytprøver:</u>

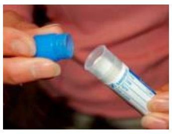

Tag en eller to af de markerede Salviette spytopsamlingrør og put begge vat-stykker i munden uden at røre med fingrene. Når vat-stykkerne har været i munden i mindst 2 minutter kan du spytte dem tilbage i rørene og sætte dem i køleskabet. Se yderligere instruktioner på det udleverede ark. Mål evt. temperatur samtidig med spytprøven.

<u>Mundtermometer:</u>

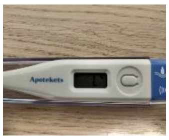

Du kan evt. måle mundtemperatur samtidig med du tager spytprøver. Tænd termometeret. Når displayet blinker med ”$L^{C°}$” er det klart til at måle. Placer termometerspidsen i munden under tungen så langt tilbage i munden som muligt, lad termometeret måle i ca. 90 sekunder, indtil det bipper. Tag det ud af munden og aflæs displayet, gør gerne dette tre gange og nedskriv den højeste værdi i ” Skema over prøvetagningstidspunkter”.

<u>Maddagbog (digitalkamera)</u>

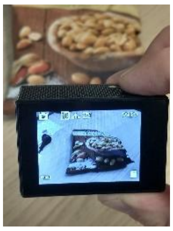

Vi vil bede dig dokumenterer alt du spiser og drikker over de to dage. Du behøves ikke veje maden, men vi vil bede dig tage billeder med det udleverede digitalkamera, alle billeder får et automatisk dato- og tidsstempel, så du behøver heller ikke notere tidspunkt. **Brug gerne samme størrelse tallerken til alle måltider. Gem gerne indpakninger eller tag billede af deklarationen.**
Du betjener kameraet ved først at trykke på tændknappen ⏻ på forsiden af kameraet. Kameraet kan skiftes mellem *billedetagning, video* og *hukommelse*. Du skifter mode ved at trykke (uden at holde) på tændknappen ⏻ igen.

Kameraet tre modes: video Billedetagning Hukommelse

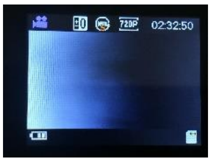


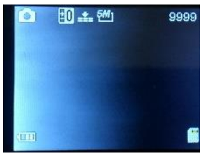


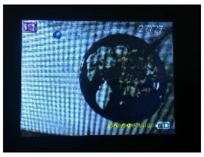

Brug billedetagning til at dokumentere hvad du spiser og drikker. Når kameraet står på ”billedetagning” tager du et billede ved at trykke på knappen øverst på kameraet (se billede i panelet til højre).

Hvis du har nogen spørgsmål kontakt Mikkel Kolind på tlf. 23 81 80 10

# Sampling Miniguide (translated)

Circadian Rhythm Disorders in Individuals with Mental Disorders

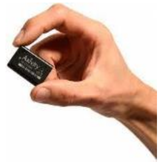

Motion Sensors (Accelerometers)

The motion sensors measure your sleep patterns and overall physical activity level over seven days. They automatically track everything and do not require any action on your part. It is important not to remove the accelerometer before the seven days have passed.

Both accelerometers start: Date:_____ / _____ Time:_____: _____ (starts automatically)
Both accelerometers are finished: Date: _____ / _____ Time: ____ : _____ (stops automatically)

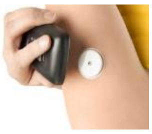

Glucose Meter

The glucose meter measures changes in your blood sugar. It should be scanned approximately every 6 hours, with the most important scan being right when you wake up.
The glucose meter is started: Date: _____ / _____ Time: _____ : _____
The glucose meter is finished: Date: _____ / _____ Time: _____ : _____

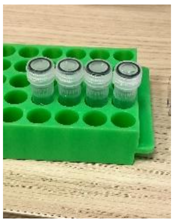

Hair Samples:

When taking hair samples, only the follicle near the root contains mRNA. It is therefore important to remove the hair with as much root as possible. If you cannot see any of the oily sheath around the root, the hair is likely broken too high and cannot be used. You should pluck approximately 20 hairs per sample.
Once you have plucked a hair, cut the hair root into one of the mRNA tubes. Ensure that the clipped hair root is covered with liquid. Only the hair root can be used for analysis; the hair shaft should not go into the liquid.

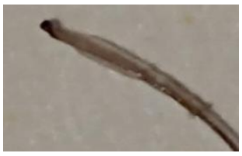

"Here you see the entire hair root. If none of the oily sheath is visible, the hair is likely broken too high, and you will need a new sample

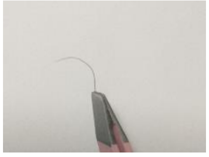

This hair appears to be broken too high (no visible hair root) and un-fortunately cannot be used

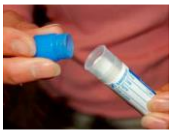

Saliva samples:

Take one or two of the saliva collection tubes and place the cotton piece in your mouth without touching it. Once the cotton pieces have been in your mouth for at least 2 minutes, you can spit them back into the tubes and put them in the refrigerator. See additional instructions on the provided Saliva guide. You may measure temperature simultaneously with the saliva sample.

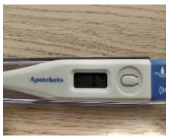

Mouth Thermometer:

You could measure mouth temperature while taking saliva samples. Turn on the thermometer. When the display blinks with "LC°," it is ready to measure. Place the thermometer tip in your mouth under the tongue as far back as possible, let the thermometer measure for about 90 seconds until it beeps. Remove it from your mouth and read the display. It's recommended to do this three times and record the highest value in the 'Sampling Time Schedule".

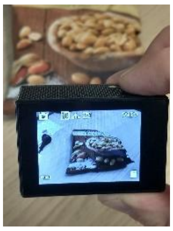

Food Diary (Digital Camera)

We would like you to document everything you eat and drink over the course of two days. You don't need to weigh the food, but we ask you to take pictures with the provided digital camera. All pictures will automatically receive a date and time stamp, so there's no need to note the time. **Please use the same-sized plate for all meals. You may save packaging or take pictures of product labels.**
To operate the camera, start by pressing the power button ⏻ on the front of the camera. The camera can switch between video recording, photo capture, and memory modes. Change the mode by pressing (without holding) the power button again.

The three modes: video Photo Capture Memory

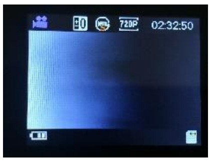


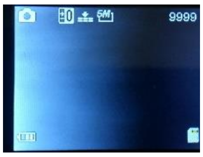


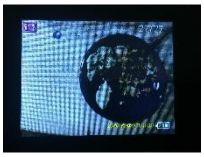

Use the photo capture mode to document what you eat and drink. When the camera is set to 'photo capture,' take a picture by pressing the button on the top of the camera

Hvis du har nogen spørgsmål kontakt Mikkel Kolind på tlf. 23 81 80 10

# Instruktion til Spytopsamling

Undgå at børste tænder, spise eller drikke noget – udover vand - 30 minutter inden prøveopsamling.

På rørets låg er der en talværdi som fortæller hvornår røret bør bruges. Et 1-tal indikerer at røret er til dagen første prøve, et 2-tal at det er til dagens anden prøve (6 timer senere) osv. Rør til hver dag ligges i separate poser.

Instruks til prøvetagning:

1. Find Salivette røret mærket med det aktuelle opsamlingstidspunkt.

2. Fjern den blå prop, og svampen kommer til syne. Inder-røret med svampen må ikke fjernes.

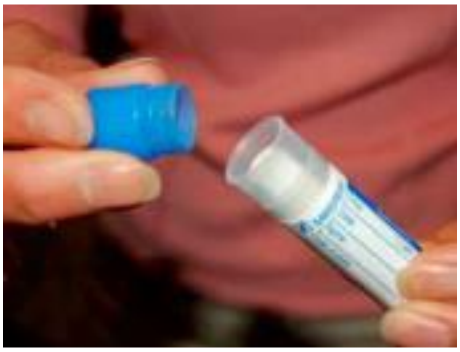

3. Tag Salivette røret op til munden og vip røret, så svampen falder ind i munden. NB – *hvis* du er blevet instrueret i at tage 2 svampe i munden bør begge puttes i munden samtidig.

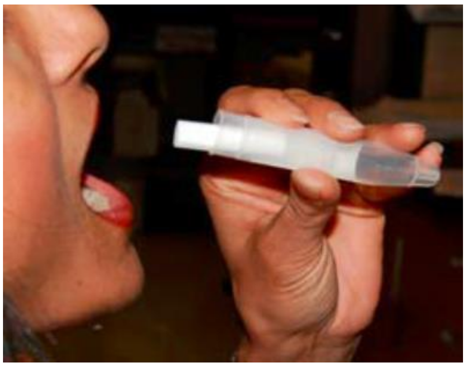

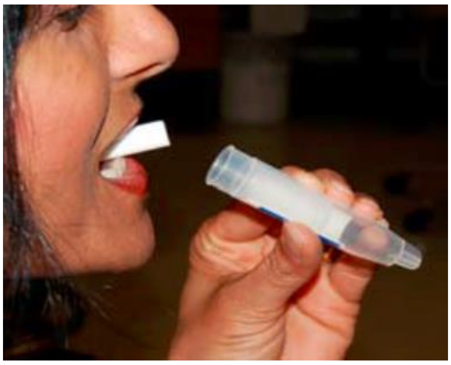

4. Når svampen er i munden: For at stimulere spytproduktionen trykkes svampen forsigtigt mellem tænderne, samtidig med at svampen forsigtigt køres rundt i munden. Svampen bør blive i munden i minimum 2 minutter
5. Spyt svampen tilbage i røret. Det gør ikke noget hvis lidt ekstra spyt kommer med ud. Forsegl herefter røret med den blå prop – vær sikker på at proppen sidder helt på. Herefter placeres røret i køleskabet.

Du kan med fordel måle din temperatur (bagerst, under tungen) mens du tager spytprøver.

Hvis du har spørgsmål er du velkommen til at kontakte daglig projektleder Mikkel Kolind på tlf.:23 81 80 10

Tak for din medvirken ☺

# Instructions for Saliva Collection

Avoid brushing your teeth, eating, or drinking anything - except for water - 30 minutes before sample collection.
On the cap of the tube, there is a numerical value indicating when the tube should be used. A 1 indicates that the tube is for the first sample of the day, a 2 indicates it's for the second sample of the day (6 hours later), and so on. Tubes for each day should be placed in separate bags

Instruks til prøvetagning:

1. Locate the Salivette tube marked with the current collection time.

2. Remove the blue cap, revealing the sponge. Do not remove the inner tube with the sponge.

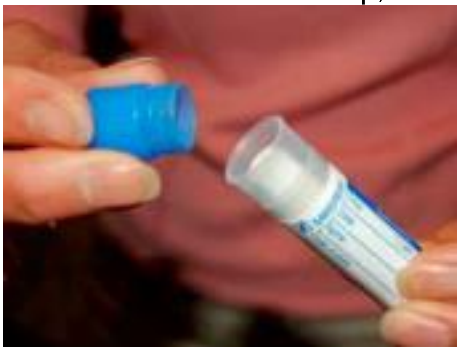

Hold the Salivette tube up to your mouth and tilt the tube so that the sponge falls into your mouth. Note - *if* you have been instructed to place 2 sponges in your mouth, both should be placed in the mouth simultaneously.

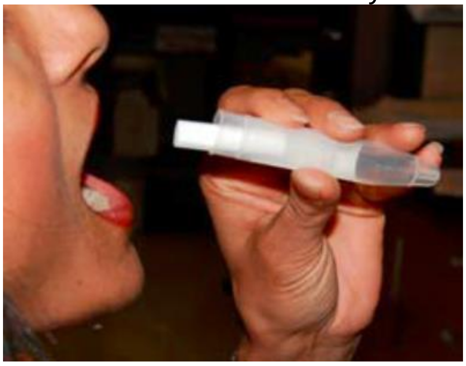

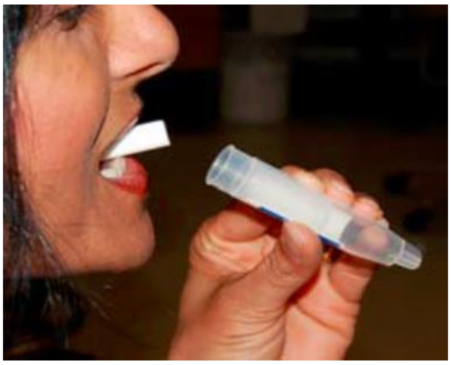

3. Once the sponge is in your mouth: To stimulate saliva production, gently press the sponge between your teeth while also gently moving it around in your mouth. The sponge should remain in your mouth for a minimum of 2 minutes.
4. Spit the sponge back into the tube. It's okay if a little extra saliva comes out. Seal the tube with the blue cap - make sure the cap is securely in place. Then, place the tube in the refrigerator..

You can also measure your temperature (at the back, under the tongue) while taking saliva samples.

If you have any questions, please feel free to contact the project manager, Mikkel Kolind, at phone number: 23 81 80 10.

Thank you for your participation! ☺

**Disclaimer:**

The translated manuals are provided for methodological transparency purposes only. Only Danish versions are utilized in the SOMBER trial. Any images included within are free to be used or redistributed without any associated licensing or restrictions.

ID:_____ Dato:______

# Skema over prøvetagningstidspunkter –
## Døgnrytme-forstyrrelser hos personer med psykiske lidelser

Normalt tidspunkt for at stå op

Kl:_________ (noteres af medarbejder, spørgsmål tidligere stillet i andet batteri)

Normalt tispunkt for at gå i seng:

Kl:_________ (noteres af medarbejder, spørgsmål tidligere stillet i andet batteri)

Der er aftalt hjemmebesøg _____dag d.____/_____ -____ kl:_____og ____dag d.____/_____ -____ kl:______

Vi vil gerne have at du tager temperaturmålinger samt spyt og hår-prøver på tidspunkterne angivet i nedenstående skema (sæt evt. alarmer/påmindelser på din telefon for at hjælpe med at huske tidspunktet):

| DAG 1 : ______dag d.___________ | | | | | | | | | | Noter temperatur: |
|---|---|---|---|---|---|---|---|---|---|---|
| | Hårprøve | Udført? | | Spytprøve | Udført? | | Mundtemperatur | Udført? | | |
| Står op | Kl | Ja | nej | Kl | Ja | nej | Kl | Ja | nej | |
| | Kl | Ja | nej | Kl | Ja | nej | Kl | Ja | nej | |
| | Kl | Ja | nej | Kl | Ja | nej | Kl | Ja | nej | |
| | Kl | Ja | nej | Kl | Ja | nej | Kl | Ja | nej | |
| | Kl | Ja | nej | Kl | Ja | nej | Kl | Ja | nej | |
| | Kl | Ja | nej | Kl | Ja | nej | Kl | Ja | nej | |
| | Kl | Ja | nej | Kl | Ja | nej | Kl | Ja | nej | |
| Går i seng | kl | Ja | nej | kl | Ja | nej | kl | Ja | nej | |

Evt. noter dag 1:_____________________________________________________________________________
_____________________________________________________________________________________________
_____________________________________________________________________________________________

| DAG 2 : ______dag d.___________ | | | | | | | | | | Noter temperatur: |
|---|---|---|---|---|---|---|---|---|---|---|
| | Hårprøve | Udført? | | Spytprøve | Udført? | | Mundtemperatur | Udført? | | |
| Står op | Kl | Ja | nej | Kl | Ja | nej | Kl | Ja | nej | |
| | Kl | Ja | nej | Kl | Ja | nej | Kl | Ja | nej | |
| | Kl | Ja | nej | Kl | Ja | nej | Kl | Ja | nej | |
| | Kl | Ja | nej | Kl | Ja | nej | Kl | Ja | nej | |
| | Kl | Ja | nej | Kl | Ja | nej | Kl | Ja | nej | |
| | Kl | Ja | nej | Kl | Ja | nej | Kl | Ja | nej | |
| | Kl | Ja | nej | Kl | Ja | nej | Kl | Ja | nej | |
| Går i seng | kl | Ja | nej | kl | Ja | nej | kl | Ja | nej | |

Evt. noter dag 2:_____________________________________________________________________________
_____________________________________________________________________________________________
_____________________________________________________________________________________________

Bemærk: Hvis tidspunktet for hvornår du faktisk står op på test-dagene afviger med mere end 30 minutter bør prøvetagningstidspunktet tilrettes. Kontakt daglig projektleder Mikkel Kolind på tlf.: 23 81 xx xx

ID:_____ Dato:______

# Schedule of Sampling Times–

## Circadian Rhythm Disturbances in Individuals with Mental Disorders

Normal time to wake up

At: _________ (noted by staff, question previously asked in another battery)

Normal time to go to bed:
At: _________ (noted by staff, question previously asked in another battery)

Home visit scheduled on ___day the___ /____ - at:_____ and ____day the ____/____ - at:______

We would like you to take temperature measurements as well as saliva and hair samples at the times indicated in the schedule below (you may set alarms/reminders on your phone to help remember the time):

| Day 1 : _____day the___________ | | | | | | | | | | Temperature: |
|---|---|---|---|---|---|---|---|---|---|---|
| | Hair sample | done? | | Saliva | done? | | temperature | Done? | | |
| Wake up | Kl | yes | no | Kl | yes | no | Kl | yes | no | |
| | Kl | yes | no | Kl | yes | no | Kl | yes | no | |
| | Kl | yes | no | Kl | yes | no | Kl | yes | no | |
| | Kl | yes | no | Kl | yes | no | Kl | yes | no | |
| | Kl | yes | no | Kl | yes | no | Kl | yes | no | |
| | Kl | yes | no | Kl | yes | no | Kl | yes | no | |
| | Kl | yes | no | Kl | yes | no | Kl | yes | no | |
| Bed time | kl | yes | no | kl | yes | no | kl | yes | no | |

notes for day 1:____________________________________________________________________________
______________________________________________________________________________________________
______________________________________________________________________________________________

| Day 2 : _____day the___________ | | | | | | | | | | Temperature: |
|---|---|---|---|---|---|---|---|---|---|---|
| | Hair sample | done? | | Saliva | done? | | temperature | Done? | | |
| Wake up | Kl | yes | no | Kl | yes | no | Kl | yes | no | |
| | Kl | yes | no | Kl | yes | no | Kl | yes | no | |
| | Kl | yes | no | Kl | yes | no | Kl | yes | no | |
| | Kl | yes | no | Kl | yes | no | Kl | yes | no | |
| | Kl | yes | no | Kl | yes | no | Kl | yes | no | |
| | Kl | yes | no | Kl | yes | no | Kl | yes | no | |
| | Kl | yes | no | Kl | yes | no | Kl | yes | no | |
| Bed time | kl | yes | no | kl | yes | no | kl | yes | no | |

notes for day 2:____________________________________________________________________________
______________________________________________________________________________________________
______________________________________________________________________________________________

Note: If the time you actually wake up on test days deviates by more than 30 minutes, the sampling time should be adjusted. Contact daily project manager Mikkel Kolind at tel.: 23 81 xx xx
