# Supplementary material for: Investigating the role of obesity, circadian disturbances and lifestyle factors in people with schizophrenia and bipolar disorder: Study protocol for the SOMBER trial
Source: PLoS One. 2024 Jul 8;19(7):e0306408. doi: 10.1371/journal.pone.0306408 (PMC11230533; doi:10.1371/journal.pone.0306408)
Supplement: S2 File — (PDF) [file pone.0306408.s002.pdf]

Navn på deltager________________________

CIVIL STATUS

Boform

☐Bor alene

☐Bor sammen med andre

Antal børn:___________

Hjemmeboende børn:______

Forsørgelse:

☐ Arbejdsløs, kommentar:_______

☐ Pensioneret, kommentar:________

☐ I arbejde

Hvis ja: ☐ deltid ☐ fuldtid ☐flex, timer___ /uge

Skifteholdsarbejde ☐ notes:_______________

Anden karakteristika:

Hårfarve:_____________

Etnicitet:______________

Gamer - notes:_____________

UDDANNELSESBAGGRUND

Højest opnåede uddannelse

☐ Folkeskole

☐ Ungdomsuddannelse (Fx, HF, Gymnasium)

☐ Mellemlang uddannelse (fx sygeplejerske, lærer, tømrer)

☐ Lang uddannelse (universitetsuddannelse)

Participant's name________________________

CIVIL STATUS

Living arrangement

☐ Living alone

☐ Living with others

Number of children:___________

Children living with you:________

Means of support:

☐ Unemployed, comment:_______

☐ Retired, comment::________

☐ Employed

If yes: ☐ part-time ☐ Fulltime ☐Flex, hours___ /week

Shift work ☐ - notes:________________

Other characteristics:

Hair colour:_____________

Ethnicity:______________

Gamer - notes:_____________

EDUCATIONAL BACKGROUND

Highest achieved education

☐ Elementary school

☐ Secondary education (e.g., HF, High School)

☐ Medium-length education (e.g., nurse, teacher, carpenter)

☐ Higher education (university degree)

	Ja	Nej	Mistænker	Ved ikke
Type 2 diabetes				
Type 1 diabetes				
Diabetes, men jeg kender ikke typen				
Forhøjet blodtryk				
Forhøjet kolesterol				
Tidligere blodprop i hjertet				
Hjertearytmi (f.eks. forkammerflimmer)				
Hjertesvigt				
Hjerte-kar sygdom				
Fedtlever				
Skrumplever				
Polycystisk Ovariesyndrom (PCOS)				
KOL (kronisk obstruktiv lungesygdom)				
Obstruktiv Søvnapnø				
Slidgigt i fodled				
Slidgigt i knæ				
Slidgigt i hofte				
Slidgigt i ryggen				
Depression				
Angst				
Skizofreni				
Bipolar lidelse				

	Yes	No	I suspect	Don’t know
Type 2 diabetes				
Type 1 diabetes				
Diabetes, but I don't know the type				
High blood pressure				
High cholesterol				
Previous blood clot in the heart				
Cardiac arrhythmia (e.g., atrial fibrillation)				
Heart failure				
Cardiovascular disease				
Obesity				
Cirrhosis of the liver				
Polycystic Ovary Syndrome (PCOS)				
COPD (chronic obstructive pulmonary disease)				
Obstructive sleep apnoea				
Arthritis in the shoulder				
Arthritis in the knee				
Arthritis in the hip				
Arthritis in the back				
Depression				
Anxiety				
Schizophrenia				
Bipolar disorder				

Kære deltager
Vi vil i følgende stille dig nogle spørgsmål vedrørende dit søvnmønster og din søvnkvalitet. Det er vigtigt at alle spørgsmål besvares. Tag dig god tid og vælg de svarkategorier som bedst passer til din situation. Din besvarelse er fortrolig og deles ikke med andre systemer eller personer end dem tilknyttet forsøget.

SOVEMEDICIN OG STIMULANSER

A) Bruger du noget medicin eller andre former for hjælpemidler for at sove bedre?

Nej, sæt kryds:_____ og gå til ”B)”
Ja, udskrevet af læge eller købt på apotek
Hvad:____ Hvor ofte:______________________
Hvad:____ Hvor ofte:______________________
Ja, lovlige håndkøbsprodukter, alkohol eller lignende
Hvad:____ Hvor ofte:______________________
Hvad:____ Hvor ofte:______________________
Ja, skaffet uden om traditionelle markeder (illegale stoffer, cannabis og lignende)
Hvad:____ Hvor ofte:______________________
Hvad:____ Hvor ofte:______________________

B) I løbet af en normal hverdag **(ikke fredag),** Hvor meget kaffe (med koffein) drikker du ca.?

antal kopper:__________
På hvilket tidspunkt af døgnet vil du normalt stoppe med at drikke kaffe?:__________

C) I løbet af en normal hverdag **(ikke fredag),** Hvor meget alkohol drikker du ca.?

antal genstande:__________
hvordan vil dit indtag af alkohol normalt fordele sig over en hverdag (angiv antal genstande):
Morgen, antal:_____ Middag, antal:____ Eftermiddag, antal:_____ Aften, antal:______

Nat, antal:_____

Har du nogen kommentarer eller uddybninger til nogle af overstående spørgsmål/svar:

____
____
____

Kære deltager
We will next ask you some questions regarding your sleep pattern and sleep quality. It is important that all questions are answered. Please take your time and select the answer categories that best match your situation. Your response is confidential and will not be shared with any systems/registries or persons other than those affiliated with the project.

SLEEP MEDICATION AND STIMULANTS

A) Do you use any medicine or other aids to help you sleep better?
No, tick here:_____ and go to "B)"
Yes, prescribed by a doctor or bought at a pharmacy
What:____ How often:______________________
What:____ How often:______________________
Yes, legal over-the-counter products, alcohol, or similar
What:____ How often:______________________
What:____ How often:______________________
Yes, obtained outside of traditional markets (illegal drugs, cannabis, and similar)
What:____ How often:______________________
What:____ How often:______________________

B) During a normal weekday **(not Friday)**, how much coffee (with caffeine) do you drink approximately? number of cups:_________
At what time of the day do you normally stop drinking coffee?:__________

C) During a normal weekday **(not Friday)**, how much alcohol do you drink approximately?

number of units:_________

How would your intake of alcohol normally be distributed over a weekday (state number of units):

Morning, amount:_____ Noon, amount:____ Afternoon, amount:_____ Evening, amount:______

Night, number:_____

Do you have any comments or elaborations to any of the above questions/answers:
____
____
____
____

After the questionnaire on sleep medications and stimulant, the participant will fill out the Pittsburg sleep quality questionnaire(PSQI).

Disclaimer:

The translated questions provided above are for the purpose of methodological documentation and to ensure transparency. They are direct translations and may not fully convey the nuances and contextual meanings inherent in the original Danish language.
