# Supplementary material for: Investigating the role of obesity, circadian disturbances and lifestyle factors in people with schizophrenia and bipolar disorder: Study protocol for the SOMBER trial
Source: PLoS One. 2024 Jul 8;19(7):e0306408. doi: 10.1371/journal.pone.0306408 (PMC11230533; doi:10.1371/journal.pone.0306408)
Supplement: S3 File — (PDF) [file pone.0306408.s003.pdf]

Manual for mounting of body worn accelerometers in the SOMBER trial

Two Axivity AX3 accelerometers (Axivity Ltd., Newcastle upon Tyne, United Kingdom) are mounted on the participants body. The Axivity AX3 is an affordable, waterproof accelerometer with built-in temperature and light measurement. The Axivity AX3 is open-source, and all software and hardware documentation are publicly available.

Step 1 - Initializing the accelerometers:

Ensure the acceloromter is sufficiently charged (>85%) and properly initialized before mounting. Accelorometers are initialized using the open-source software OMGui. Software for initialization and downloading can be found on the following website: https://github.com/digitalinteraction/openmovement/wiki/AX3-GUI

Once downloaded perform the following steps:

1. Start [AX3] OMGui software.

2. Connect both instruments to the computer (via USB).

3. Wait for both instruments to appear on the Device list.

4. If there is data on the instruments, download the data if it hasn't already been done.

5. Ensure that the instrument is empty. Use "Clear" to delete the contents.

6. Select both instruments from the list.

7. Activate "Record" to initialize the instruments, a dialog box should open (see picture 1).

8. Set Sampling Freq (Hz) to 50Hz, Range (g) to 8.

9. Specify Recording time (when the instrument should start and stop recording):

a. Select "Interval."

b. First, indicate when the instruments should start recording. Chose the first full hour after the participant is officially enrolled in the study.

c. For the recording duration, you can either specify a "Duration" or a specific date/time. The duration should be 8 whole days (192 hours)

11. Make sure "Unpacked Data" is active/selected.

12. Click the "Ok" button.

13. In the "Device" list, you can see how the accelerometers have been initialized. Ensure that you have selected the correct interval.

14. Remove the accelerometer from the computer (USB).

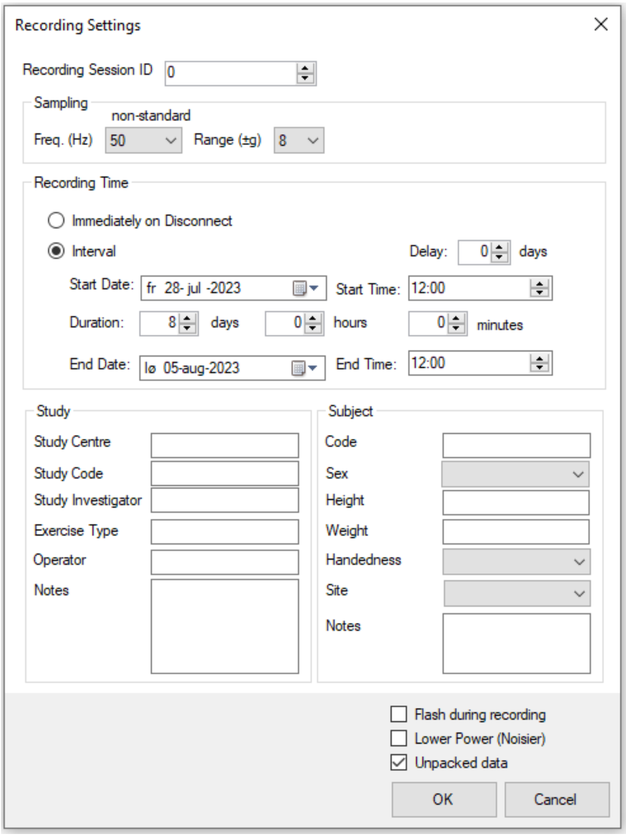

Picture 1 – initializing the accelerometer in OMGui

The Axivity AX3 instrument has a sharp edge from the factory on the opposite end of the USB plug. In order to avoid skin perforation or shearing, these edges can be rounded of using sandpaper to remove a small amount of edge material.

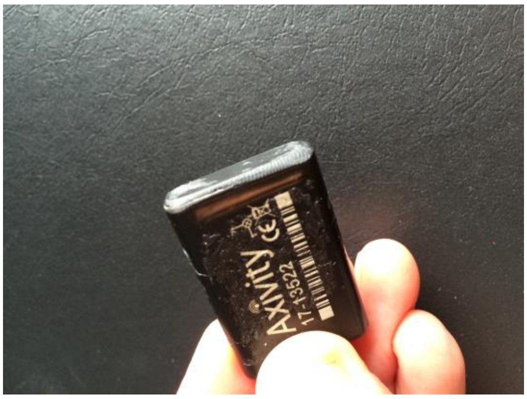

Picture 2 - Edges on the accelerometer are smoothed to avoid skin perforation

Step 2 – preparing and mounting the thigh accelerometer.

The accelerometer is mounted on the thigh using three different types of tape, and a an optionel thin sealing film. The steps below details the procedure for preparing the accelerometers. Any generic tape brand may be used. However, for all tape in direct contact with the skin, medical grade tape with no skin-irritants (allergy-friendly) and permeable, allowing sweat/fluid to be transported from the skin to the surroundings.

Suggested products:

Sealing film: Parafilm® 100 mm x 38 mtr (Hounissen, Skanderborg, Denmark). link: https://www.hounisen.com/laboratorieudstyr/forbrugsartikler/papirvarer/parafilm-og-dispenser/parafilm-100-mm-x-38-mtr

Top tape: Opsite flexifix, 10m x 5 cm, (Smith+Nephew, Watford, United Kingdom)

http://www.smith-nephew.com/professional/products/advanced-woundmanagement/opsite/opsite-flexifix/

Double-sided adhesive tape: brand type not important. Any strong thin double adhesive tape will do. This tape will not touch any skin areas. Double sided carpentry tape works well. Example link:

http://dk.rs-online.com/web/p/dobbeltsidet-plasttape/5034884/

Bottom tape: Fixomull stretch 10 m x 5 cm (BSN Medical, Hamburg, Germany). Link:

https://kruuse.com/da-dk/produkter/op-site-flexifix-5-cm-x-10-m?GroupID=

With the items ready follow these steps:

1. (Optional) wrap the accelerometer in sealing film (see picture 3). This makes it so that cleaning the accelerometers after use become much easier (the acceleromters should be cleaned between uses and avoiding sticky glue residue make this a much faster process – this does not influence data gathering.

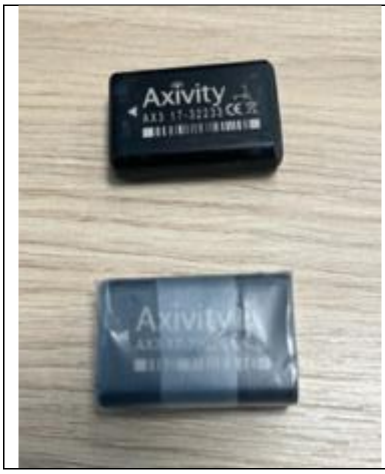

Picture 3: accelerometers wrapped in sealing film (Parafilm®) (bottom). Unwrapped accelerometer (top)

2. Prepare a piece of Fixomull stretch tape a piece of double-sided tape. The Fixomull tape should be slightly larger than the instrument itself, approximately 5 millimeters on all sides. The double-sided tape should not be larger than the instrument.

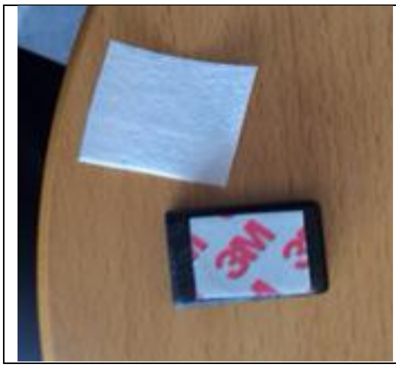

Picture 4: Fixomull before attaching to accelerometer

3. Prepare the top tape. Cut one or two pieces of the Opsite flexifix, dependent on the tape width. We used the 5cm width and therefor cut two pieces. Round the corners of the tape pieces – this will make it less likely that a corner of the tape comes lose (picture 6).

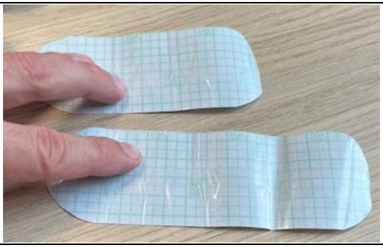	*Picture 5: Opsite flexifix with rounded corners*

4. Mount the double-sided tape on the back of the instrument (where there is no text) (picture 4).

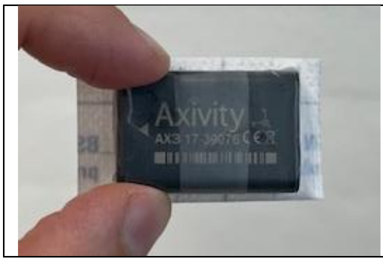	*Picture 4 – The double adhesive is placed on the back of the accelerometer (where there is no serial number). Using the double adhesive, the bottom tape is then glued to the accelerometer with the adhesive side of the bottom tape facing upward. When turned around the serial number should face upwards and the adhesive side downwards.*

5. After cleaning (and if necessary shaving) the thigh area, the AX3 is placed on the thigh at the mid-point between knee and hip and mid between the lateral and medial border of the thigh, with the USB port towards the knee and the text facing upwards. After securing the accelerometre in this position with the Fixomull (bottom tape). The top-tape (Opsite flexifix) is applied, first in the transverse direction and then in the longitudinal direction.

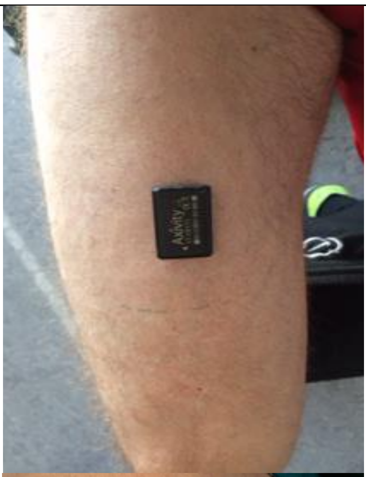	*Picture 5 – position of the accelerometer on the thigh*
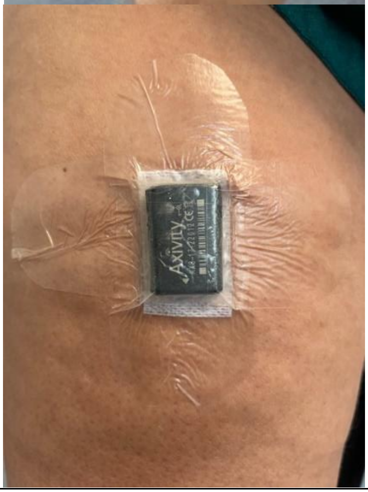	*Picture 6 –accelerometer on the thigh after applying all tape*

Step 3 – preparing and mounting the wrist accelerometer..

Wrist placement

The accelerometer should be placed in the Axivity bracelet (Axivity Ltd., Newcastle upon Tyne, United Kingdom). The small direction indicators (arrows) should be visible and face in the same direction. The bracelet is then placed on the participants non-dominant wrist similarly to an ordinary wristwatch (picture 7C).

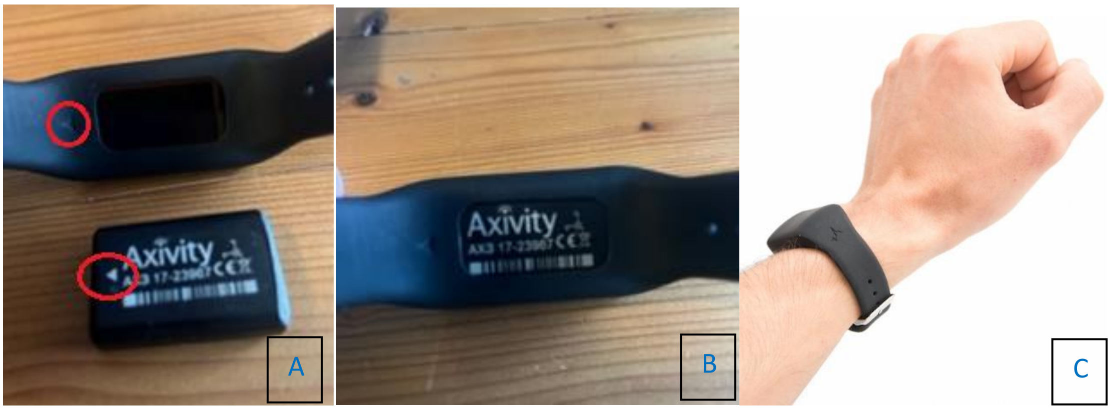

Picture 7 A,B,C – The accelerometer placement in the bracelet. The direction indicators (circled in red) should face the same direction (A), with the serial number facing downwards towards the wrist when placed (B). The bracelet is then placed on the non-dominant wrist and worn like an ordinary wristwatch (C).

Concluding the visit

The participants are given the final instruction and handed the “mini-guide” manual, where start and stop time of the accelerometers are noted.
