# Supplementary material for: Investigating the role of obesity, circadian disturbances and lifestyle factors in people with schizophrenia and bipolar disorder: Study protocol for the SOMBER trial
Source: PLoS One. 2024 Jul 8;19(7):e0306408. doi: 10.1371/journal.pone.0306408 (PMC11230533; doi:10.1371/journal.pone.0306408)
Supplement: S4 File — (PDF) [file pone.0306408.s004.pdf]

Noter, skemaer og tjeklister til hjemmebesøg 1&2

Besøg 1:

ID:_______

Dato:________

Tidspunkt:________________

**Selv-tests:**			Noter:
Modtaget spytprøver	☐ja	☐nej	
Modtaget hårprøver	☐ja	☐nej	
Temperatur angivet	☐ja	☐nej	
**Prøvetagning med hjælp fra testleder:**			
Spytprøver	☐ja	☐nej	
Hårprøver	☐ja	☐nej	
Temperatur	☐ja	☐nej	

Var prøverne opbevaret i køleskabet?

☐ja ☐nej

Hvad tid stod deltageren op?

Kl. ___: ______ Hvor lang tid mellem prøveindsamling og stå op? ____t :____m

Hvad tid gik deltageren i seng?

Kl. _____: _____ Hvor lang tid mellem prøveindsamling og sengetid? ____t :____m

Notes, forms, and checklists for home visits 1&2

Visit 1:

ID:_______

Date:________

Time:________________

Self-tests			Notes:
Received saliva samples	☐yes	☐no	
Received hair samples	☐yes	☐no	
Temperature noted	☐yes	☐no	
**Sampling with assistance from the test leader:**			
Saliva	☐ja	☐nej	
Hair	☐ja	☐nej	
Temperatur	☐ja	☐nej	

Were the samples stored in the refrigerator?

☐Yes ☐ No

What time did the participant get up?

Time. ___: ______ How long between sample collection and getting up? ____t :____m

What time did the participant go to bed?

Time. _____: _____ How long between sample collection and bedtime? ____t :____m

Kvalificering af digital maddagbog – dag 1

ID:________

Dato:_________

Tidspunkt:________________

Prøver deltageren på at tabe sig?

Ja ☐ nej ☐

Mener deltageren at den registrerede kost afspejler et normalt dagsindtag for ham/hende?

Ja ☐ nej ☐ Evt. note:____________________

Billede tids-stempel	note

Qualification of digital food diary – day 1

ID:_______

Date:________

Time:________________

Is the participant trying to lose weight?

Yes ☐ No ☐

Does the participant believe that the registered diet reflects a normal daily intake for him/her?

Yes ☐ No ☐ Possible note:___________________

Photo timestamp	notes

Hjemmebesøg dag 1 ID:_______ dato:________

Lys i soveværelset

1) Når du har slukket lyset I dit soveværelse, er der så stadig nogen synlig lyskilde?

ja☐ nej☐

Hvis du svarede "Ja": Vil du beskrive lyskilden som kunstigt (eks. Lofts- eller standerlampe) eller naturlig (eks, sol, stjerne eller måne) eller er der både kunstig og naturlig belysning?

kunstig☐ naturlig☐ både kunstig og naturlig belysning ☐

2) Var du i stand til at se nogen genstande i dit soveværelse?

ja☐ nej☐

Hvis du svarede "Ja": fremstod de genstande du kunne se gråtonede eller i farver? (gælder også mindre levende farver)

Gråtonede ☐ i farver ☐

Hvilke af følgende har du i dit soveværelse?

- PC/Laptop ja☐ nej☐
- Fjernsyn ja☐ nej☐
- Smartphone ja☐ nej☐
- Tablet ja☐ nej☐

Brug af medie-enheder og skærmtid

Angiv i nedenstående skema hvor lang tid du bruger med forskellige medieenheder. Tidsforbrug omfatter kun tid hvor skærmen er tændt og hvor du kan se hvad der sker på skærmen. Eksempelvis hvis du lytter til en lydbog på din smartphone mens den ligger i lommen skal dette ikke tælles med.

	Før kl 12		12-18	18-21	Efter 21	
Hvor lang tid har du brugt på følgende i minutter?	Morgen (i sengen før du står op)	Morgen/formiddag (efter du er stået op)	Middag og eftermiddag	aften	Sen aften/ nat (før du går i seng)	Nat (efter du er gået i seng)
PC/Laptop						
Fjernsyn						
Smartphone						
Tablet						

Home visit day 1 ID:_______ date:________

Lighting in the bedroom

3) When you have turned off the light in your bedroom, is there still any visible light source?

Yes ☐ No ☐

If you answered "Yes": Would you describe the light source as artificial (e.g., ceiling or floor lamp) or natural (e.g., sun, stars, or moon), or is there both artificial and natural lighting?

Artificial ☐ Natural ☐ Both artificial and natural lighting ☐

4) Were you able to see any objects in your bedroom?
Yes ☐ No ☐

If you answered "Yes": Did the objects you could see appear in grayscale or in color? (this also applies to less vivid colors)

Grey-scale ☐ In color ☐

Which of the following do you have in your bedroom?

- PC/Laptop Yes☐ No ☐
- Television Yes ☐ No ☐
- Smartphone Yes ☐ No ☐
- Tablet Yes ☐ No☐

Brug af medie-enheder og skærmtid

Please indicate in the following chart how much time you spend with different media devices. Time usage includes only time when the screen is on and you can see what is happening on the screen. For example, if you are listening to an audiobook on your smartphone while it is in your pocket, this should not be counted.

Time of day	Before 12 noon		12-18	18-21	After 21	
How long have you spent on the following in minutes?	Morning (in bed before getting up)	Morning/forenoon (after getting up)	Noon and afternoon	Evening	Late evening/ night (before going to bed)	Night (after going to bed)
PC/Laptop						
Television						
Smartphone						
Tablet						

The Above example is a translation of the protocol for visit 1 – the procedure and questions for day 2 are identical.

The translated protocol is provided for methodological transparency purposes only. Only Danish versions are utilized in the SOMBER trial.

The questionnaires on light in the sleeping environment and media device usage are modified from Harrison et al (Harrison, Yablonsky, Powell, Ancoli-Israel, & Glickman, 2019) and Green et al (Green, Barak, Shine, Kahane, & Dagan, 2020)

References

Green, A., Barak, S., Shine, L., Kahane, A., & Dagan, Y. (2020). Exposure by males to light emitted from media devices at night is linked with decline of sperm quality and correlated with sleep quality measures. *Chronobiology International, 37*(3), 414-424.

Harrison, E. M., Yablonsky, A. M., Powell, A. L., Ancoli-Israel, S., & Glickman, G. L. (2019). Reported light in the sleep environment: enhancement of the sleep diary. *Nature and Science of Sleep*, 11-26.
