# Supplementary material for: Investigating the role of obesity, circadian disturbances and lifestyle factors in people with schizophrenia and bipolar disorder: Study protocol for the SOMBER trial
Source: PLoS One. 2024 Jul 8;19(7):e0306408. doi: 10.1371/journal.pone.0306408 (PMC11230533; doi:10.1371/journal.pone.0306408)
Supplement: S5 File — (PDF) [file pone.0306408.s005.pdf]

main supplementary variables

1. Sleep parameters (actigraphy)
    a. Sleep length – time from sleep onset to offset (in minutes)
    b. Sleep quality – ratio of total sleep time to time spent in bed measured (as percentage)
    c. Sleep variability – individual variation in total sleep time across all days (as SD)
    d. Sleep variability – individual variation in sleep onset time (as SD)
    e. Sleep variability – individual variation in sleep offset time (as SD)
2. Apnoea-severity (Cardio-respiratory monitoring with Nox T-3)
    a. Oxygen desaturation index (events per hour)
    b. Apnoea-hypopnea index (events per hour)
    c. Time snoring during sleep (as percentage of sleep time)
    d. Total Apneas and Hypopneas: Number of apneas and hypopneas recorded (total count).
    e. Obstructive Apneas (OA): Number of obstructive apneas per hour of sleep (events per hour).
    f. Mixed Apneas (MA): Number of mixed apneas per hour of sleep (events per hour).
    g. Central Apneas (CA): Number of central apneas per hour of sleep (events per hour).
    h. Hypopneas: Number of hypopneas per hour of sleep (events per hour).
    i. Obstructive Hypopneas (OH): Number of obstructive hypopneas per hour of sleep (events per hour).
    j. Central Hypopneas (CH): Number of central hypopneas per hour of sleep (events per hour).
    k. Respiratory Disturbance Index (RDI): Total number of apneas, hypopneas, and Respiratory Effort-Related Arousals (RERAs) per hour of sleep (events per hour).
    l. Hypoventilation: Occurrence and duration of hypoventilation during sleep (events per hour).
    m. Respiration Rate: Average number of breaths per minute.

n. Average Oxygen saturation (SpO2)

o. Min SpO2 during sleep

p. Min pulse during sleep

q. Max pulse during sleep

r. Average pulse during sleep

3. Body composition (Bio impedance)

    a. Percent Body Fat: Proportion of weight that is fat.

    b. Dry Lean Mass: Weight of lean mass excluding water.

    c. Body Fat Mass: Total weight of body fat.

    d. Skeletal Muscle Mass: Weight of muscle attached to bones.

    e. Basal Metabolic Rate: Calories needed at rest for physiological functions.

    f. Lean Body Mass: Body weight minus fat mass.

    g. Body Mass Index: Ratio of weight to height squared.

    h. Total Body Water: Total water content in the body.

    i. Segmental Lean Analysis: Lean mass in body segments (arms, legs, trunk).

    j. Visceral Fat Level: Estimated fat around internal organs.

    k. Extracellular Water: Water outside body cells.

    l. Intracellular Water: Water inside body cells.

    m. Segmental Fat Analysis: Fat mass in body segments.

    n. Muscle-Fat Analysis: Comparison of muscle to fat mass.

    o. Fitness Score: Assessment of fitness based on muscle and fat.

    p. Body Balance Assessment: Balance of muscle and fat distribution.

4. Body composition (Clinical measure)

    a. Waist to hip circumference

5. Physical activity level (actigraphy)

a. Average accelerometre counts per day (total counts)

b. Average time spend in sedentary activity (% per day)

c. Average time spend in light activity (% per day)

d. Average time spend in moderate to vigorous physical activity (% per day)

e. Average peak time for moderate to vigorous physical activity

6. Diet and meal timing (food diary)

   a. Dietary distribution of macro nutrients (% fat, %carbohydrates, %protein)

   b. Micronutrient analysis – percentage of participants with indication for micronutrient deficits

   c. time between first and last meal

   d. Midpoint between first and last meal

7. Psychopharmaceuticals (self-report and from registries)

   a. Dichotomous variable (used/not used)

   b. Therapeutic class, e.g., antipsychotics, mood stabilizers, antidepressants ect.

   c. Duration of medication use

   d. Dosage of medication use

      i. Converted, when applicable, to chlorpromazine equivalent units for antipsychotics
      ii. Converted, when applicable, to diazepam equivalent units for anxiolytics.

   e. Polypharmacy: Number of psychopharmaceuticals used.

8. Light exposure

   a. Self-reported light in the sleeping environment categorized as complete darkness, low light or some light.

   b. Time between last media device usage and sleep initiation

   c. Total time using media devices (any)

   d. Total time using smartphone

   e. Total time using Television

f. Total time using tablet

9. Continuous glucose monitoring
   a. Average Glucose Level: Mean glucose level over a 24-hour period.
   b. Glucose Variability: Standard deviation of glucose levels, indicating the degree of fluctuation.
   c. Time in Range: Percentage of time glucose levels are within the target range.
   d. Time Above Range: Percentage of time glucose levels are above the target range.
   e. Time Below Range: Percentage of time glucose levels are below the target range.
   f. Postprandial Glucose Peaks: Maximum glucose levels observed after meals.
   g. Nocturnal Glucose Levels: Average glucose levels during sleep hours.
10. Body temperature
    a. Oral temperature as 8 individual measures per participant
    b. Oral temperature minimum
    c. Oral temperature maximum
    d. Oral temperature average
    e. Surface temperature (thigh mounted accelerometer) – time of sleep minimum
    f. Surface temperature (thigh mounted accelerometer) – minimum
    g. Surface temperature (thigh mounted accelerometer) – maximum
    h. Surface temperature (thigh mounted accelerometer) – average
11. Physical function
    a. Handgrip strength – highest of 3 measures (in kg)
    b. 1-leg standing balance (in seconds)
    c. 10-meter usual gait speed (m/s)
    d. 10-meter maximal gait speed (m/s)
12. Self-reported sleep quality (Pittsburg sleep quality index)

a. Subjective Sleep Quality: Assessment of the individual's perception of their sleep quality.
b. Sleep Latency: Amount of time it takes to fall asleep after going to bed (in minutes)
c. Sleep Duration: Total amount of actual sleep time in a night (in minutes)
d. Habitual Sleep Efficiency: Ratio of sleep duration to the total time spent in bed.
e. Sleep Disturbances: Frequency and types of sleep disturbances (e.g., waking up in the middle of the night, bathroom trips).
f. Use of Sleeping Medication: Frequency of using medication to aid sleep.
g. Daytime Dysfunction: Degree to which sleep problems affect daily functioning (e.g., staying awake during daytime activities).

13. Chronotype (Morning-eveningness questionnaire)
    a. Total score
    b. Grouped: Definite Evening Type, Moderate Evening Type, Intermediate Type (Neither Type), Moderate Morning Type, Definite Morning Type.
14. Use of caffeinated drinks
    a. Total amount of caffeine (self-report)
    b. Total amount of caffeine (documented in food diary)
    c. Time from last caffeinated drink to sleep initiation (self-report)
    d. Time from last caffeinated drink to sleep initiation (documented in food diary)
15. Other variables
    a. Hair color
    b. Ethnicity
    c. Living arrangement (alone, together, with/without kids)
    d. Oral Ph
16. Patient reported difficulty self-sampling. (Ordinal 0-10 scale. 0 = not troublesome at all, 10 = very troublesome.)

a. Difficulty hair sampling (0-10)

b. Difficulty saliva sampling (0-10)

c. Difficulty keeping food diary (0-10)

d. Difficulty wearing and using continuous glucose monitor (0-10)

e. Difficulty wearing accelerometer (0-10)

f. Optional comments or notes to each measure

g. Optional comments or notes on participating in the project as a whole.
