# Supplementary material for: Investigating the role of obesity, circadian disturbances and lifestyle factors in people with schizophrenia and bipolar disorder: Study protocol for the SOMBER trial
Source: PLoS One. 2024 Jul 8;19(7):e0306408. doi: 10.1371/journal.pone.0306408 (PMC11230533; doi:10.1371/journal.pone.0306408)
Supplement: S7 File — (PDF) [file pone.0306408.s007.pdf]

Protocol revision history

Protocol version 3.4, 19-11-2023 , approved November 23, 2023)
Extension of study period end date from January 2024 to December 2024

Protocol version 3.3, 17-05-2023, approved September 15, 2023
Amendment: Introduction of a new recruitment pathway (Psychinfo collaboration)

Protocol version 3.2, 13-01-2023, approved January 23, 2023
Amendment: Introduction of new recruitment pathway (healthy controls through social media postings)

Protocol version 3.1, 13-04-22, approved May 12, 2022
Minor corrections to participant information and protocol.
Minor corrections to questionnaires and sampling guides

First Ethical approval

Protocol version 3.0, 08-03-2022, approved march 24th 2023
